# Omicron: a challenge to hybrid immunity – correspondence

**DOI:** 10.1097/JS9.0000000000000268

**Published:** 2023-03-13

**Authors:** Ankush K. Niranjan, Shailesh K. Patel, Talha B. Emran

**Affiliations:** a Departments of Veterinary Microbiology; b Veterinary Pathology, College of Veterinary Science and Animal Husbandry, Rewa, Madhya Pradesh, India; c Department of Pharmacy, BGC Trust University Bangladesh, Chittagong; d Department of Pharmacy, Faculty of Allied Health Sciences, Daffodil International University, Dhaka, Bangladesh

Highlights

- Primarily, adaptive immunity plays a pivotal role.
- Immunological memory is the key to combating re-infection.
- T cells and B cells develop a wider range of antibodies.
- The ongoing coronavirus disease 2019 vaccination protocol follows intramuscular administration.

*Dear Editor,*

The immune system, an interwoven network tailored within the body to encounter foreign invaders, has the ultimate goal of defending against infections. Primarily adaptive immunity plays a pivotal role and induces antigen-specific immunological memory through exposure to either natural infection or vaccination. Antigen-specific immunological memory cells proliferate upon subsequent antigen exposure and rapidly generate effector molecules. Hence, immunological memory is the key to combat re-infection, embedding the role of T-lymphocyte and B-lymphocyte during primary encounter. The development of the immunological memory depends on the antigens encountered, time frame, and frequency of exposure. Memory B cells are a heterogeneous population of cells that the body generates in response to an infection, which plays a role in the generation of homologous antibodies during re-infection and supports the encoding of a repository of immunological variants. On the other hand, to develop a wide variety of memory B cells, help from T cells is essential. So together, T cells and B cells develop a wider range of antibodies effective against the infectious variants 1.

In the context of the global pandemic coronavirus disease 2019; the immunity is induced through vaccination (artificial), infection (natural), or both. In response to the recurrent wave of severe acute respiratory syndrome coronavirus 2 (SARS-CoV-2) infection and rigorous vaccination strategies (irrespective of SARS-COV-2 variants) majority of the population developed hybrid immunity (inducted through infection and vaccine). Hybrid immune individuals had more frequent exposure to SARS-CoV-2 antigens than those with either vaccination or infection alone.

The rise in the neutralizing antibody titer above the threshold defines the immune response to the infection or vaccination. Vaccination after SARS-CoV-2 infection augments the number of persistent clones and recruits additional B-cell clones to the memory pool. The SARS-CoV-2 memory T cells and B cells rise in the presence of the Wuhan-Hu-1 strain or its Alpha, Delta, and Beta variants. Cross-variant neutralizing antibodies are produced in great quantities by vaccine-induced memory B-cell production and potently amplified by SARS-CoV-2 infection, providing protection against re-infection for a long period of time 2. The hybrid immunity addresses more effectively the cross-variant neutralization approximately 100-folds than the infection and 25-folds than the vaccination alone, until the rise of Omicron. Furthermore, it also provides an umbrella against the more severe and frequent infections 3.

At present, it seems like hybrid immunity is the best tactic against the SARS-CoV-2 but, despite, the vaccination (most population vaccinated by the vaccine derived from the early SARS-CoV-2 variants namely, Alpha, Beta, Gamma, and Delta), mutation in the spike proteins evolved the Omicron variants and its sublineages which have low pathogenicity, confinement to upper respiratory tract, and generation of weak immune response. This raise the concern as spike proteins are the only antigen expressed by various mRNA and virus-vectored vaccines, despite the fact that the body is exposed to all viral antigens during infection. The ongoing coronavirus disease vaccination protocol follows intramuscular administration and generates systemic immunoglobulin (Ig)G, whereas SARS-CoV-2 infection of the upper respiratory tract induces mucosal IgA. The upper respiratory tract often produces substantially less systemic IgG following infection than other parts of the body. Individuals who have been immunized in response to infection or vaccination may be less likely to practice preventative measures such as wearing a mask, isolating themselves from others and washing their hands often; hence, they are more vulnerable to the recent omicron variants, as they escape the neutralizing antibodies generated in response to vaccination. The effectiveness of hybrid immunity is frequently dependent on the recipient’s response to the administered vaccination and the gradual decline of neutralizing antibodies over the time.

To develop and implement efficient preventative measures, it is essential to understand the magnitude of neutralizing titers and the breadth of T-cell responses to both known and unknown SARS-CoV-2 variants. To successfully improve pre-existing cross-protective antibody titers mediated by hybrid immunity, vaccine candidates encompassing conserved areas of SARS-CoV-2 variants other than the spike protein may be used as a booster. By stimulating pre-existing resident immune cells in the mucosa, vaccination at a mucosal surface promotes better systemic and mucosal immunity than intramuscular injection, and may be especially useful for persons with hybrid immunity. Even after vaccination and recovery from infection, individuals could face a high risk of contracting Omicron (and future) variants; thus, it is important to strictly adhere to the guidelines of preventive measures, namely, avoiding unnecessary contact with others, wearing a mask, isolating themselves from others, washing hands regularly, etc., especially for old and immune-compromised people.

## Ethical approval

Not applicable.

## Sources of funding

No funding was received.

## Author contribution

A.K.N.: conceptualization, data curation, writing – original draft preparation, writing – reviewing and editing. S.K.P.: data curation, writing – original draft preparation, writing – reviewing and editing. T.B.E.: writing – reviewing and editing, visualization, supervision.

## Conflicts of interest disclosure

Authors declare that they have no conflicts of interest.

## Research registration unique identifying number (UIN)

1. Name of the registry: not applicable.
2. Unique identifying number or registration ID: not applicable.
3. Hyperlink to your specific registration (must be publicly accessible and will be checked): not applicable.

## Guarantor

Talha Bin Emran, PhD, Associate Professor, Department of Pharmacy, BGC Trust University Bangladesh, Chittagong 4381, Bangladesh. Tel: +880 303 356 193, Fax: +880 312 550 224. https://orcid.org/0000-0003-3188-2272.

## Data statement

The data in this correspondence article is not sensitive in nature and is accessible in the public domain. The data is therefore available and not of a confidential nature.

## Provenance and peer review

Not commissioned, internally peer-reviewed.
